# Thrombocytopenia and thrombosis in hospitalized patients with COVID-19

**DOI:** 10.1186/s13045-020-01003-z

**Published:** 2020-12-01

**Authors:** Heng Mei, Lili Luo, Yu Hu

**Affiliations:** 1 Institute of Haematology, Union Hospital, Tongji Medical College, Huazhong University of Science and Technology, 1277 Jiefang Avenue, Wuhan, 430022 China; 2 Hubei Clinical and Research Centre of Thrombosis and Hemostasis, Wuhan, China

**Keywords:** COVID-19, Thrombocytopenia, Thrombosis

## Abstract

As our understanding on coronavirus disease 2019 (COVID-19) deepens, it is increasingly recognized that COVID-19 is more than a respiratory condition. Thrombocytopenia and thromboembolic complications are a composite factor associated with critical COVID-19 and increased mortality. Immune-inflammation-mediated destruction, severe acute respiratory syndrome coronavirus 2 (SARS-CoV-2) infection per se and increased consumption are proposed to be responsible for thrombocytopenia. Multiple concomitant conditions or results caused by SARS-CoV-2 infection are high risk factors for thrombosis. Recently, platelet activation and platelet-mediated immune inflammation induced by SARS-CoV-2 infection were also found to be the contributors to the thrombosis in COVID-19 patients. In addition to thrombus scoring system, D-dimer is an excellent indicator for monitoring thrombosis. COVID-19 patients with high risk for thrombosis should be subjected to early thromboprophylaxis, and prolonged activated partial-thromboplastin time should not be a barrier to the use of anticoagulation therapies in the control of thrombosis in COVID-19 patients.

Starting in December 2019, coronavirus disease 2019 (COVID-19), which is caused by severe acute respiratory syndrome coronavirus 2 (SARS-CoV-2), has become a public health crisis around the world. As the seventh known human coronavirus genus, SARS-CoV-2 is comparable to SARS-CoV and Middle East respiratory syndrome coronavirus in that they all cause unusual viral pneumonia. As we gain further insights into the condition, COVID-19 is more a systemic condition than it is a respiratory disease, especially in severe cases. Mounting evidence has revealed that thrombocytopenia and thromboembolic complications are associated with disease severity and increased mortality [1, 2].

## Phenomenon and mechanisms of thrombocytopenia in COVID-19

A national multicentre retrospective study conducted in China revealed that the incidence of thrombocytopenia (< 150 * 10^9^/L) on admission in COVID-19 was 36.2% [3], which is similar to that in SARS (40–45%%) and MERS (36%) [4]. It has been widely accepted that thrombocytopenia is indicative of disease severity, and a progressive decline of platelet counts was significantly associated with increased mortality [1]. Although thrombocytopenia is often believed to be an indicator of bleeding, the frequency of bleeding events was reportedly significantly lower in COVID-19 than in Ebola and other infections caused by haemorrhagic fever viruses. Liao et al*.* found that only three out of 55 nonsurvivors experienced nonlethal haemorrhagic events [4]. Bowles et al*.* also showed that no clinically significant haemorrhage was found in 35 COVID-19 patients who suffered from a prolonged activated partial-thromboplastin time (aPTT) [5]. The possible reason for this might be that the pattern of coagulation disorder in COVID-19 is of a more severe hypercoagulative state rather than a hypocoagulative one [6]. Currently, the mechanisms by which SARS-CoV-2 causes thrombocytopenia are speculated to involve the following: (1) an impaired haematopoietic microenvironment caused by systemic inflammation or cytokine storm, for example, elevated IL-6, which is a common phenomenon in SARS-CoV-2 infection [4], could suppress haematopoiesis [7]. (2) SARS-CoV-2 might directly infect haematopoietic stem cells or megakaryocytes through angiotensin-converting enzyme 2 (ACE2), CD13 or CD66a, as in other coronavirus infections that elicit thrombocytopenia [8]. (3) antiviral antibodies cross-reacting with haematopoietic cells and (or) platelets, e.g. anti-adenovirus antibodies, can cross-react with platelet integrin GPIIb/IIIa [8]. Indeed, Chen et al*.* showed that delayed-phase thrombocytopenia was the result of impaired maturation of megakaryocytes in COVID-19 patients [9]. (4) An autopsy of nonsurvivors revealed thrombotic microangiopathy and disseminated intravascular coagulation, which lead to the increased consumption of platelets [6]. (5) Activated platelets can be scavenged via splenic/hepatic macrophages. In fact, two separate teams independently provided evidence that platelets are hyperactivated in COVID-19 patients [10, 11]. The activation of the Mitogen-activated protein kinase pathway could partially explain this platelet hyperreactivity [10, 11].

Similar to the influenza virus, rhinovirus and some other viruses, SARS-CoV-2 may also directly interact with platelets, thereby altering their quantity and/or function. ACE2 has been shown to be a receptor of both SARS-CoV-2 and SARS-CoV. SARS-CoV-2 possesses an affinity for ACE2 that is at least 10 times greater than that of SARS-CoV [12]. Transmembrane protease serine 2 (TMPRSS2), a serine protease, mediates the SARS-CoV-2 virus cell membrane fusions by proteolytically cleaving and activating the spike protein. Recently, Zhang et al*.* discovered that platelets expressed abundant ACE2 and TMPRSS2 [11]. Here, SARS-CoV-2 was shown to directly induce platelet activation and aggregation and promote thrombosis [11].

## Thrombosis and thromboprophylaxis in COVID-19

Accumulating evidence has shown that thromboembolic complications emerging from COVID-19 are one of the main reasons for sudden deterioration and death [13]. Several independent teams reported that the incidence of thromboembolic events in severe patients and nonsurvivors was much higher than in their nonsevere counterparts and survivors, even though thromboprophylaxis was administered [2, 14].

Helms et al*.* showed that compared with non-COVID-19 patients who suffered from acute respiratory distress syndrome (ARDS), COVID-19 patients with ARDS had significantly more thrombotic events (11.7% vs. 4.8%), mainly pulmonary embolism (11.7% vs. 2.1%) [14]. Autopsies confirmed that nonsurvivors of COVID-19 were filled with thrombi and microthrombi in multiple organs [15]. The thromboembolic lesions in COVID-19 were predominantly in the lungs, principally red thrombi formed by a fibrin network and red blood cells, which differed from white thrombi, the main types of thrombi in SARS [15].

Multiple factors or mechanisms have been proposed to account for thromboembolic events. Many concomitant conditions or results caused by SARS-CoV-2 infection are high-risk factors for thrombosis (those in arteries, veins or microthrombosis), including advanced age, obesity, coexisting chronic diseases (including diabetes, cardiovascular disease, etc.), fever, dehydration, inevitable prolonged bed-ridden state, invasive treatment, endothelial injury, refractory hypoxemia, hyperinflammation and others, which encourage vascular occlusion because of hypercoagulability, hypoperfusion and vasoconstriction [4, 11, 13].

Platelets play a crucial role in thrombogenesis. Most recently, elevated platelet activation, including platelet aggregation, platelet spreading, α granule secretion and dense granule release, among others, was proved to be closely linked to thrombosis in COVID-19 [11]. Moreover, Zhang et al*.* exhibited that SARS-CoV-2 and its spike protein directly stimulated platelets, resulting in the release of coagulation factors and inflammatory cytokines and enhancing leukocyte–platelet aggregates, which can promote thrombosis and thrombus stability [11].

Early detection and timely thromboprophylaxis can lower the incidence of thrombosis. Zhang et al*.* demonstrated that thromboprophylaxis halved the incidence of deep-vein thrombosis in patients with COVID-19, with the Padua prediction scores being 4 or higher [2]. Dynamic monitoring of D-dimer and platelet counts, as well as the use of thrombus scoring systems, such as Autar, Caprini, Padua and Improve, are believed to be of value for assessing the risk of thrombosis [13]. Indeed, in a retrospective study, a multivariate logistic regression analysis showed that D-dimer elevation was an excellent in predicting thrombosis [2]. As a general consensus for thromboembolism prevention, appropriate hydration and proper physical therapy should be provided for severely ill patients. All hospitalized COVID-19 adult patients should receive appropriate anticoagulation unless the risk of bleeding outweighs the danger of thrombosis (abnormal prothrombin time or aPTT). Low molecular weight heparin is recommended with higher priority for thromboprophylaxis than unfractionated heparin. When not available or having contraindications of heparin anticoagulant drugs, non-heparin anticoagulant drugs, such as argatroban or bivalirudin, are recommended [13]. In patients contraindicated for anticoagulant agents, mechanical preventive measures are recommended [13]. The precise timing, type and dose of preventive anticoagulation therapy remain unclear.

## Conclusion

Thrombocytopenia and thrombotic complications in COVID-19 patients are common and contribute to a higher mortality rate. Significant bleeding events are rarely reported in COVID-19. Dynamic monitoring, early detection and thromboprophylaxis can help improve the outcome for those not contraindicated for the treatments.

## Data Availability

Not applicable.

## Abbreviations

COVID-19: Coronavirus disease 2019
SARS-CoV: Severe acute respiratory syndrome coronavirus
aPTT: Activated partial-thromboplastin time
ACE2: Angiotensin-converting enzyme 2
TMPRSS2: Transmembrane protease serine 2
ARDS: Acute respiratory distress syndrome
